# Family Meals, Conviviality, and the Mediterranean Diet among Families with Adolescents

**DOI:** 10.3390/ijerph18052499

**Published:** 2021-03-03

**Authors:** Andrea de la Torre-Moral, Sergi Fàbregues, Anna Bach-Faig, Albert Fornieles-Deu, F. Xavier Medina, Alicia Aguilar-Martínez, David Sánchez-Carracedo

**Affiliations:** 1 Faculty of Health Sciences, Universitat Oberta de Catalunya (Open University of Catalonia, UOC), 08018 Barcelona, Spain; andreadltorrem@uoc.edu; 2 Department of Psychology and Education, Universitat Oberta de Catalunya (Open University of Catalonia, UOC), 08018 Barcelona, Spain; sfabreguesf@uoc.edu; 3 FoodLab Research Group (2017SGR 83), Faculty of Health Sciences, Universitat Oberta de Catalunya (Open University of Catalonia, UOC), 08018 Barcelona, Spain; fxmedina@uoc.edu (F.X.M.); aaguilarmart@uoc.edu (A.A.-M.); 4 Food and Nutrition Area, Barcelona Official College of Pharmacists, 08009 Barcelona, Spain; 5 Eating and Weight-Related Problems Unit, Universitat Autònoma de Barcelona, 08193 Barcelona, Spain; albert.fornieles@uab.cat (A.F.-D.); david.sanchez@uab.cat (D.S.-C.); 6 Department of Psychobiology and Methodology of Health Sciences, Universitat Autònoma de Barcelona, 08193 Barcelona, Spain; 7 Department of Clinical and Health Psychology, Universitat Autònoma de Barcelona, 08193 Barcelona, Spain

**Keywords:** conviviality, family meals, Mediterranean diet, meals, adolescents, qualitative research, interviews, commensality

## Abstract

Two aspects that characterize the Mediterranean diet (MD) are “what” and “how” we eat. Conviviality relates to “how” we eat and to the pleasure of sharing meals with significant people. The most studied concept is “family meals”, which includes conviviality, which involves “enjoying” family meals. Given the lack of research on convivial family meals in Mediterranean countries, the purpose of this qualitative study was to analyze the family meal representations and practices of families with 12- to 16-year-old adolescents to assess whether they responded to a pattern of conviviality, and to examine their association with MD adherence. Twelve semi-structured interviews were conducted and food frequency and family meal questionnaires were administered. A food pattern analysis was carried out and digital photos of meals were analyzed to examine eating habits and meal composition, respectively. The findings showed that parents believed family meals are a space for socialization and communication. Items relating to the conviviality of family meals identified in the study were meal frequency, meals at the table, lack of digital distractions, pleasant conversations, and time spent on family meals. Attention should be paid to conviviality in Mediterranean families when designing multi-approach strategies to promote healthy eating among adolescents.

## 1. Introduction

The Mediterranean diet (MD) is considered one of the healthiest dietary patterns in the world because of its observed association with positive health outcomes [1]. After more than half a century of research, the MD has been recognized by UNESCO [2] as an intangible cultural heritage of humanity [3], including the dimension of shared meals or commensality in its definition as a symbol of cultural heritage. Two aspects that characterize the MD are “what” and “how” we eat [4]. The characteristics of the MD have been represented using a pyramid [4] that not only summarizes the dietary pattern, but also portrays food-related cultural and social activities and lifestyle aspects, including the regular practice of physical activity, culinary activities, and conviviality. Conviviality is the social component of eating involving the sharing of meals, also contributing to the associated health-promoting effects of this lifestyle [5]. Conviviality is a feature in Mediterranean countries where family and food traditions are core activities [6].

Conviviality is more than a shared meal, because it relates to “how” we eat. The “how” involves socializing when eating and, as complementary aspects, self-awareness of satiety and hunger signals, eating slowly, and chewing well [7]. Meal satisfaction is not only related to portion sizes or how much is eaten [8]. The activity of cooking, conviviality, and the fact of sitting together to eat the same foods rather than just eating are very typical of the Mediterranean areas and are habits practiced on a daily basis [9]. In the Mediterranean lifestyle, conviviality has been related to the pleasure of sharing meals [8]. Sharing meals with family and friends (significant people) establishes a sense of community and contributes to the perpetuation of the MD pattern from generation to generation [5].

As far as we know, conviviality has not been studied in the literature of Northern European and English-speaking countries. Family meals, on the other hand, defined as “to eat meals together as a family on a daily basis” [10,11,12], is a common research topic on adolescent risk prevention and healthy development, including its relation to a better dietary intake [13,14,15,16]. However, these meals do not have to be convivial in the established sociological sense of the term. The term “family meal” encompasses conviviality, which should be considered a more specific term, as it also entails the idea of enjoying family meals.

Several qualitative studies have highlighted the multiple benefits and barriers associated with family meals [17], which affect their frequency and quality. Family connection and communication are the main elements linked to shared meals [17], since this is seen as a space for socializing and promoting togetherness [18,19,20,21,22,23]. Adolescents believe that the preparation of tasty, home-cooked meals can help improve the quality of family meals [19]. While using technological devices such as mobile phones and watching television during meals might have a negative effect on communication [24] and the quality of the interaction between family members [21,24], lack of time [25], tiredness, and timetable conflicts [17] have likewise been identified as barriers to sharing meals among families. However, there is a lack of research focused on the features, benefits, and challenges of convivial family meals in Mediterranean countries.

Within the MD pattern, the “what” can be described as the quantities, proportions, and frequencies of foods consumed, or the components defining a diet and lifestyle pattern. Food pattern analysis through indices or scores has been used to study the synergistic effect of the overall combination of foods [26]. The MD pyramid provides guidance on overall diet, while a healthy plate guide indicates the proportions of food to eat per meal (suggesting that half a plate should be filled with fruits and vegetables) [27,28]. Adhering to the MD has been related to higher nutritional adequacy and a better nutrient profile with a lower prevalence of individuals showing inadequate intakes of micronutrients [29]. The use of fresh and minimally processed plant-based foods typical of the MD implies cooking [30], and convivial family meals have a protective group effect leading to less overeating [31]. Both factors may also directly or indirectly connect people and hunger–satiety signals, which could help in maintaining body weight in Western societies facing the obesity pandemic [32]. Traditional dietary patterns have shifted towards a more “westernized” dietary pattern that relies to a greater extent on fast foods and ready-to-use ultra-processed food products [30,33], especially in the younger generations [30]. Furthermore, with urbanization, globalization, and modern lifestyles, progressively fewer meals are being eaten together with the associated loss of the social component of the meal [34].

Conviviality in the MD, however, has different characteristics to family meals, such as the enjoyment of sharing a table and food with significant people. To date, conviviality has only been studied from a theoretical perspective, while empirical approaches to examining this cultural attribute of the MD are lacking. Given the social and geographical particularities of Mediterranean countries, there is a need to gain a better understanding of families’ views of their experiences and practices of conviviality. This qualitative study explored family meal representations and practices in a sample of Mediterranean families with 12- to 16-year-old adolescents to assess whether they respond to a pattern of conviviality and their association with MD adherence, thus filling the gap in the literature.

## 2. Materials and Methods

### 2.1. Study Design

A qualitative descriptive approach [35] was used in this study to describe the perspectives and practices of families regarding conviviality. This approach is especially suited to research situations where little is known about the phenomenon of interest, requiring an inductive approach that prioritizes the participants’ voices and experiences above the researchers’ perspective [36]. Qualitative description involves a low level of interpretation of the studied events to improve the descriptive validity of the study [37], especially when the research questions focus “on discovering the who, what, and where of events or experiences” [38]. In this study, the use of qualitative description enabled the authors to stay close to the data, minimize research bias, and develop a rich, straightforward and accurate understanding of the topic of conviviality based on the participants’ words spoken in their own natural setting. At the same time, the fact that different sets of methods used in qualitative food research present their own characteristics, perspectives, and applications was taken into account [39].

### 2.2. Sampling

Criterion sampling was used to identify two-parent families resident in Catalonia (Spain) with at least one adolescent child between the ages of 12 and 16 years. Potential participant families were selected by identifying their composition. Several formal and informal identification strategies were used, including the following: (a) asking parent associations and teachers to send emails to potential contacts (including information on the study purpose, what participation would entail, and how to contact the study team should they be interested); (b) asking acquaintances to help identify potential participants; (c) snowballing from families that had already participated in the study. Potential participants were initially contacted by telephone or email to assess eligibility and to arrange a schedule of interviews for the candidates considered eligible. Participants were interviewed until thematic saturation was achieved on the basis that no new themes were identified by the researchers in further interviews. Saturation was assessed by the first author by reading the interview transcripts, leading to the recruitment of 12 participant families. This sample size is consistent with the guidelines suggested in the literature on qualitative sampling [40,41]. Table 1 shows the characteristics of the 12 participating families. Most of the families had a high socioeconomic status with both parents with a university education and full-time jobs. The interviewed parent was usually the mother. The adolescents living in the home were aged between 14 and 16 years, and their weight was within normal range in most cases.

### 2.3. Data Collection

Twelve semi-structured interviews [42] with either the mother (*n* = 10) or both parents (*n* = 2) were carried out in the families’ homes between December 2019 and January 2020. Each participating family was interviewed once. The adolescent children were not present during the interview to avoid their possible influence on the parents’ responses. The interviews lasted between 20 and 30 min and were conducted in Spanish or Catalan by A.D. The interviews were audio-recorded and transcribed verbatim by an external transcription service. All the transcripts were reviewed for accuracy. Follow-up probes and prompts were used to encourage interviewees to expand on their answers. The interview guide, which was generated based on the research questions and key themes of the literature review, included open-ended questions on their representations and practices regarding family meals, including the following sets of topics: the organization of the daily routine of family meals, where meals are eaten, the importance of family meals, the use of technology while eating, and the type of conversations that occur during family meals (see Supplementary Table S1 for the interview guide). Parents were asked to complete a food frequency questionnaire (FFQ), an adaptation of Pastor et al. 2017 [43], to assess food consumption. They also answered a questionnaire on family meals based on the EAT project [44] and the results from different qualitative studies on the subject, which included questions on the frequency of family meals (breakfast, lunch, and dinner), the average number of minutes spent on family meals, the frequency of use of electronic devices during family meals, and the frequency of actively watching screens during family meals. Moreover, families were asked to take photos of the food consumed during family meals over three days, considering that a family meal is one that is shared by all the family members living together. The adolescents’ self-reported height and weight were requested. This study was conducted according to the guidelines laid down in the Declaration of Helsinki, all procedures were approved by the Institutional Review Board of the Universitat Autònoma de Barcelona (No. 3451), and a signed informed consent form was obtained from the participants before the interview.

### 2.4. Data Analysis

Data-driven thematic analysis was performed on the transcribed interviews, as described by Boyatzis [45]. This type of analysis is particularly suited to qualitative descriptive designs, since the themes are extracted from the participants’ views rather than previously defined by the researchers. The analysis was carried out in three steps by three members of the research team (A.D., A.B., and S.F.). In the first step, A.D. selected and repeatedly read a subsample of four interviews. The main themes identified in the interviews were summarized and compared to identify common themes, which were converted into codes that were associated with a label, a definition, inclusion and exclusion criteria, and coding examples. The structure and content of the coding scheme was then discussed with A.B. and S.F. to verify that the codes properly represented the summarized themes.

Disagreements between the researchers were resolved through discussion, and any required changes were made. In the second step, the interview transcripts were coded by A.D. using the coding scheme and assisted by the software NVivo version 12 (QSR International, 2020). Passages of data related to the study research questions or topics were systematically coded on a line-by-line basis. The coding was then scrutinized by A.B. and S.F. In the third step, a reiterative approach was used by A.D. to sort, collate, and combine the codes into overarching themes, which were then examined by A.B. and S.F. to ensure that they were relevant to the research questions or topics. All members of the research team agreed on the final list of themes. Last, the Nvivo matrix coding query function was used to identify patterns in the data across participants. This process helped to establish within-case generalizability and interpretive validity [46].

The families’ dietary patterns were assessed using two scores (the Mediterranean diet pyramid score and the Mediterranean diet adherence screener (MEDAS)), while the FFQ data and meal composition were determined according to the healthy plate guidelines, as described below. The Mediterranean diet pyramid score was measured to determine the degree to which a family’s diet met the specific recommendations of the MD pyramid [4], similar to the healthy food pyramid score by Gila-Díaz et al. [47], as described in Supplementary Table S2. An adaptation of MEDAS was used [48], since alcohol consumption was not measured because the research took place within the family setting.

Furthermore, the food composition of the families’ plates was assessed using the 2–3 photos taken by each family in accordance with the Harvard Healthy Eating Plate proportions (vegetables 35%, fruits 15%, starchy foods 20%, proteins 20%, olive oil 5%, and water/tea/infusion 10%). Any deviation was calculated according to these percentages, a process similar to the one followed by Christoph et al. 2017 [28]. Body mass index using the adolescents’ self-reported height and weight was calculated.

## 3. Results

### 3.1. Qualitative Interviews

Two main topics arose from the interviews with families: parents’ representations of family meals and practices in family meals. Thematic analysis of the interviews revealed ten themes related to the representations and practices of conviviality in the participating families (Table 2). Following recommendations in the literature (Corden and Sainsbury, 2006 [49], Eldh et al., 2020 [50]), quotations from the study participants are presented to provide a more fine-grained illustration of the themes and to allow readers to assess the accuracy of the analysis.

#### 3.1.1. Parents’ Representations of Family Meals

Theme 1: Importance of family meals and the interactions they produce.

Parents mentioned several reasons for the importance of family meals in their everyday life. As a unifying space of socialization, family meals were a moment to share and be together as a family in a way that allowed them to create and strengthen their ties. Family meals were described by some parents (*n* = 10) as one of the few moments during the day when they were able to spend time and chat with their children.

Parents also reported that family meals were an ideal setting for educating their children and imparting cultural and social values, although disagreements between parents and their children might also occur.

*[Family meals] are a meeting place, a place for thinking about day-to-day life, about what’s happening in the city, in the country, in the world. Also, about what’s happening at home and what’s happening to them [children] outside it*.

(Family #07)

Because of this, most of the parents reported a relevance and, therefore, an enjoyment of the family synchrony and togetherness represented by these moments. They expressed satisfaction about the usefulness of these moments and that they specifically enjoyed the fact that all members of the family conversed, since the rest of the time everyone tended to go their separate ways.

*For me, [family meals] are almost paramount, because it is the time when we can communicate.*

(Family #01)

*Because in the end it’s a time of day when you find yourself. [...] But it is more of a hotel than a family. [...] Because when they’re little it’s easier, but when they get to adolescence and preadolescence there’s a disconnect between them and you. [...] These conversations, which are very spontaneous, do allow you to enter their world a little.*

(Family #11)

However, other parents (*n* = 2) reported that family meals were no more important than other activities that formed part of their daily routine. In their view, there was nothing special or distinctive about sharing a meal compared to other family activities. A few parents (*n* = 2) also reported that they were not able to enjoy family meals, as they often felt tired from work.

*Importance? [...] They [family meals] are a routine activity. They have no particular relevance*.

(Family #06)

Regarding interactions, parents (*n*=10) mentioned the importance of the conversations occurring during family meals for maintaining the bonds with their children. They felt that family meal conversations were a good opportunity for finding out more about their children’s growth and development, given that they were an ideal setting for sharing their experiences with their children while actively listening to their views and opinions. Furthermore, family meal conversations were perceived by parents as a means for strengthening family ties, as they provided a suitable setting for planning day-to-day family activities.

*I think they [family meal conversations] are a way to learn about your children. And particularly depending on their age, because when they’re younger it’s easier, but when they are adolescents there’s a distancing*.

(Family #11)

Parents (*n* = 2) who did not place any importance on family meals also assigned a low relevance to conversations occurring during meals. They talked about the unpleasantness of some of these conversations, especially when their children argued with each other.

*They [children] often disagree with each other, and I’m very tired and a bit of a zombie. But occasionally, rarely, we have nice conversations*.

(Family #04)

Theme 2: Parents’ influence on adolescents’ food preferences.

Parents (*n* = 12) highlighted their role as educational models for their children, especially regarding their dietary habits. Aware of this influence, they said they tried to ensure that the nutritional quality of family meals was high. In their view, family meals could contribute to a healthier eating experience.

*I’ve been teaching them to eat a little bit of everything since they were little, but it’s true that if you don’t like something, you don’t like it and that’s okay. So, having a meal as a family helps them to learn to eat more varied and balanced meals.*

(Family #08)

Parents (*n* = 5) used various strategies to make some foods more acceptable to their children or to encourage the consumption of new types of food, such as the use of new cooking techniques to increase the palatability. Some parents (*n* = 4) reported that they tried to camouflage the foods that their children did not like, while others (*n* = 3) simply left these foods in view in case their children felt like eating them.

*I always try to bring blueberries. The kids don’t like them, but I put them there*.

(Family #04)

#### 3.1.2. Practices during Family Meals

Theme 3: Daily meal routines.

Parents (*n* = 12) mentioned that the main factor determining the number of family meals per day was the schedule of the family members, since everyone had a different routine, the children got up at a different time to their parents to go to school, and few parents (*n* = 3) ate breakfast with their children. When it was time for lunch, many of the children (*n* = 5) ate in the school canteen. Others (*n* = 7) ate alone at home because their parents ate at work or came in later and ate lunch without them. Thus, the main meal as a family was dinner.

*So, I work in the afternoon and get home at 9:00 p.m. So it’s my partner who makes the dinner, and then, yes, we all have dinner together*.

(Family #03)

Theme 4: Location of meals.

Parents reported that family meals took place in various locations. Some families (*n* = 9) had their meals sitting at the dining room or kitchen table, while others (*n* = 3) had their meals at a coffee table by the sofa. Those who usually ate at the dining room or kitchen table sometimes had their meals on a coffee table on special occasions. Each family member was assigned a fixed place for no apparent reason, or else they rotated places every day to avoid arguments among their children.

*In the kitchen. We have a table; we have a big kitchen.*

(Family #07)

The location of non-family meals was usually the same as for family meals. Sometimes, however, these non-family meals were eaten at a coffee table or in individual rooms.

Theme 5: Use of technologies.

Regarding the use of screens (e.g., TV, mobile devices), parents (*n* = 12) reported that mobile phone use was not allowed during family meals, although there were exceptions such as for work purposes. Some families (*n* = 4) watched TV during family meals. Parents stated that television was a way of starting a conversation and that they occasionally tried to find something that all family members liked to watch. Regarding video devices, the TV was the one most used during family meals. Other parents (*n* = 8) reported that they did not allow the use of any video devices during family meals, except on special occasions such as on weekend evenings.

*No, the TV is not on. [...] We have our meals without television. No mobile phones either*.

(Family #05)

There were usually no restrictions on the use of video devices during non-family meals. In fact, some parents (*n* = 9) or children ate alone while working at the computer. Other parents (*n* = 3) said they tried not to look at any screens while eating and that they preferred to read a book or listen to the radio.

*Sometimes if they’re late my son or the older son go to the studio and have dinner in front of the computer*.

(Family #08)

Theme 6: Communication.

Parents (*n* = 11) explained that family meal conversations were often about how the children’s day or their extracurricular activities had gone, or how their friends were. Parents also talked about their work, but the conversations seemed to be more centered on their children’s day-to-day life. They usually told anecdotes or talked about their worries and problems, although some families (*n* = 2) preferred to deal with problems at other times. Other habitual topics were the household organization or what they were going to do the next day or during the week.

*[We talk about] what’s going on with them [children] each day, or anything that’s worrying them or any news they’ve heard. A little bit of everything. Or things they have to do, planning*.

(Family #05)

Parents stated that they often started the conversations by asking their children questions about how their day had gone. Many of them (*n* = 8) stated that their children were very talkative.

Theme 7: Organization.

Parents’ explanations of ways of organizing family meals were heterogeneous. Some parents (*n* = 3) commented that they planned what they would eat each week and did the shopping based on that plan. Other parents (*n* = 1) commented that they sometimes planned the menus, but they did not usually follow them. Others still reported that they did not follow any type of plan and that they improvised what they would eat each day (*n* = 8). As for the cooking of meals, some families cooked just before the meal while others did some preparation in advance, either the day before or in the morning.

*It’s very improvised. [...] My daughter sometimes eats the same thing at school as we are having for dinner at home. I wish I had time to plan, but I never do*.

(Family #03)

As for the distribution of tasks, parents described two different patterns: either the mother oversaw everything related to family feeding or else there was an equal distribution of tasks between the two parents. In the latter case (*n* = 9), the task sharing usually depended on the parents’ working hours. When a parent was not at home, the other parent took responsibility for the cooking or shopping.

*Their father generally takes care of lunch and I take care of dinner, but it’s true that we do plan a bit. [...] We prepare it, yes. The day before or in the morning*.

(Family #05)

Parents (*n* = 9) mentioned that the food was usually served at the dining-room table rather than in the kitchen. It was usually the mother who served up the food for their children, so the children did not usually choose the amount of food on their plate.

Theme 8: Meal duration.

Parents (*n*= 10) agreed that when they ate alone they ate faster than when they had family meals. Some (*n* = 2) said that they tried to read to slow the pace down or to become aware of the speed they were eating at so as not to eat too fast. They pointed out that during family meals they tended to eat slower, in a more calm and relaxed way, because talking while eating forced them to slow down.

*I think you go faster if you’re alone, right? I think so, because that’s how it is, conversation makes you take it easier*.

(Family #09)

Theme 9: Rules.

With respect to rules, other than those dealing with the use of video devices, the most common ones reported by parents (*n* = 10) were sitting up properly, everyone starting to eat at the same time, and not leaving the table or the room until everyone had finished. Parents did not mention any rules about having to finish all the food on the plate.

*There are rules. Do not get up until you’re done. Not getting up until everyone’s finished is something we haven’t accomplished yet. That’s hard. And starting all at once, if possible*.

(Family #10)

Theme 10: Dietary habits.

Parents (*n* = 9) explained that family meals usually consisted in three courses: first, main, and dessert. When the preparation was very complicated, the meal was usually just a main and a dessert. The first course was usually cooked or raw vegetables or soup. The main course usually contained a protein source such as meat, fish, or eggs. The desserts were usually fruit or yoghurt. Parents said that all family members usually ate the same food unless there was a strong aversion to certain foods or if a person was vegetarian. Some parents (*n* = 3) expressed their concerns about the degree of sophistication of the family meals, as they would have liked them to be more carefully prepared.

*As a rule, everyone eats the same thing. This is not an à la carte restaurant. We tend to repeat a lot*.

(Family #11)

The non-family meals were different from the family meals in terms of their structure. Parents mentioned that they usually made a single course, which usually entailed much less preparation, or they ate food leftovers from other days. In addition, some parents (*n* = 10) commented that they often ate less and that the meal included a more limited variety of food groups than when they had a family meal.

*It’s when I’m alone, I eat a little bit worse, you know what I mean? If I’m alone, I like eating leftovers or something fast*.

(Family #03)

### 3.2. Dietary Pattern and Meal Pattern Analysis

A dietary assessment was made using a food frequency questionnaire. Table 3 shows the Mediterranean diet pyramid scores and whether the family meal practices were employed by the families. The families were classified according to the Mediterranean diet pyramid scores for adherence to the MD. Three of the 12 families had low adherence, while the other families were classified in the “very optimal adherence” and “optimal adherence” to the MD categories. High and significant correlations between the Mediterranean diet pyramid scores and MEDAS were observed (data not shown), suggesting that both tool were appropriate for measuring adherence to the MD. The two indices classified the families in a similar way (Supplementary Table S3), although the pyramid index seemed to differentiate between families more according to their dietary habits.

The following five items contained in Table 3 also describe family meal practices: family meal frequency, meals at the table, lack of digital distractions, pleasant conversations, and time spent on meals. These items were derived from the parents’ responses to the interviews and questionnaires. Most notably, all the low adherence families failed to fulfil even one item of conviviality. The families with optimal or low adherence to the MD showed a greater tendency to consume more portions of ultra-processed foods per week. The only family with an overweight adolescent (Family #2) was the family with the lowest MD adherence score, who had a medium socioeconomic status. Moreover, the families whose meals lasted the longest also tended to have higher adherence to the MD.

In line with the parameters of the Harvard Healthy Eating Plate, the photos of the family meals showed that the proportions of the very low adherence family (Family #2) deviated the most from the Healthy Eating Plate [51] model and did not include fruit. In contrast, families with a very high adherence to the MD deviated least from the Healthy Eating Plate model and stood out especially for their high vegetable content (see Supplementary Figure S1). Regarding non-family meals, data from the interviews suggested that these tended not to follow the Harvard Healthy Eating Plate [51] proportions, as they usually consisted of sandwiches and other foods, which sometimes lacked certain food group components such as vegetables, which, according to the Harvard Healthy Eating Plate [51], should cover half the dish.

## 4. Discussion

This study presents parents’ family meal representations and practices within a Mediterranean context and provides evidence of the relationship between adherence to the MD and the practice of conviviality. The MD includes health, sociocultural, economic, and environmental factors [52], thereby stressing the role of traditions, meals, family life, and conviviality [53]. In the Mediterranean lifestyle, conviviality has been associated with the pleasure of sharing meals. Indeed, unlike other works on family meals in the literature, our study includes the enjoyment element of conviviality contextualized with its sociocultural determinants [8]. A very important aspect is the fact that these meals are shared with significant people.

Regarding parents’ views of family meals, it was highlighted that they were a space for socialization and communication and one of the few chances they had to talk to their children during the day, as pointed out by other authors [17,19,20,21,54]. To this end, the conversations during these meals were important to families, because they allowed bonds to be created between family members and were an opportunity to express opinions and listen to others. These aspects are especially relevant in adolescence when offspring tend to isolate themselves from the family environment. The parents believed that family meals gave them the opportunity to instill healthy eating habits in their offspring. It was found that it was during these regular family meals that parents consistently acted as role models for healthy eating [55,56].

Regarding specific practices in family meals, the findings from this study show that regardless of MD adherence, all the families interviewed had an optimal family meal frequency (≥5/week). In other studies, it has been found that family meal frequency has a positive effect on the growth and development [57], emotional wellbeing [58], school performance [57], and weight status [59,60] of children and adolescents. Like other family routines, meal frequency is associated with better health and wellbeing for the whole family [61] and even feelings of parenting competence [62] and family functioning outcomes [15]. Family meal times, and especially dinnertime, which has already been considered to be the main family meal [63], are one of the few moments during the day when a family can be together.

Although the previous literature has already shown that the frequency of family meals is an important protective factor, it is not entirely known why. This study contributes to the elucidation of the specific processes occurring during family meals, which in turn help to understand this observed effect. Thus, the use of technologies at meals, communication during meals, and the location of meals could play important roles. Families that did not have digital distractions during family meals tended to have a healthier dietary pattern [64]. Family communication was negatively affected by distractions, such as watching TV or using mobile devices. Only a few of the interviewed families actively watched TV regularly (not as a background), with their attention focused on the screen, thus affecting the feeling of “enjoying” the conversation, the meal, and the company. For instance, in families with optimal and very optimal MD adherences, technology was mainly used to start conversations rather than as a distraction, and to share something that also involved enjoyment.

In the interviews, parents expressed that an important issue was rules during family meals. The results revealed that families that watched TV and/or had meals at a table by the sofa did not have any other behavioral rules or had fewer in comparison to the rest of the families. In other studies, family rules have been associated with healthier outcomes related to healthy eating, sedentary behaviors, and weight status [65,66].

Regarding the duration of family meals, parents explained that non-family meals were shorter than family meals. During non-family meals, digital distractions were often present and members of the family often ate less food and had less variety of food groups on their plates, especially vegetables. As already seen, families whose meals lasted longest also tended to have higher adherence to the MD. These results support the idea that family meals improve the nutritional quality of meals [67] and satiety levels because eating fast with digital distractions decreases both [68,69]. Furthermore, the diet quality of people who eat alone is generally lower than that of people who eat together [70], and meal composition is conditioned by commensality right from the preparation stage.

The role that family meals play in terms of healthier food choices has previously been reviewed [59], showing that family meal frequency is positively related to the intake of fruits and vegetables and negatively related to sugar-sweetened beverage intake. However, there is a weaker relationship with the intake of snacks, fast food, and desserts [15]. There has been a “nutrition transition” from a Mediterranean dietary pattern to a more Western dietary pattern [71], which is high in fast food and ready-to-use ultra-processed food and low in whole grains and plant-based food. This pattern is associated with a high prevalence of obesity [72]. The results of this study are consistent with that evidence. The families with less adherence to the MD had a low consumption of fruit, whole cereals, and nuts, a high consumption of red and processed meat and sweets, and a higher frequency of ultra-processed food consumption. Moreover, the family with the least adherence had a medium socioeconomic status and one of their adolescent children was overweight. This is in keeping with other studies that state that low-income families tend towards lower adherence to the MD [31].

Thus, in light of the results of previous research, for better communication and mindful eating, there should be no distractions [54], the location should be adequate [73], a considerable amount of time should be invested in meals [72], and conversations should be pleasant [15]. Frequent family meals have been associated with a healthier dietary pattern [16]. Concurring with the above statement, we can highlight the campaign Implica’t (“Get involved”), launched by the Catalan Government’s Roundtable on the Prevention of Eating Disorders [74], and the mention of the importance of conviviality (at least one family meal a day) within the Mediterranean context from the Spanish Society of Community Nutrition [75]. Key topics that encourage and support healthy eating practices among children and adolescents are “eating together”, which highlights the link between eating socialization through regular family meals and a healthful diet, and “the pleasure of eating”, which “associates the younger generation’s healthy eating with pleasure through repeated exposure to healthful foods, enjoyable social meals, and enhancement of the cognitive qualities of healthful foods” [73].

The study findings should be considered alongside several limitations. The findings are exploratory (i.e., particular to the cases studied) and should not be generalized. Due to the purposive nature of the sample, the study participants were not representative of the universe of Mediterranean families. Consequently, the transferability of the findings to families with other cultural, educational, and socioeconomic backgrounds should be considered with caution. Socioeconomic and cultural levels have an impact on the degree of adherence to the MD for a number of reasons, including access to high-quality food, awareness of balanced lifestyles [76], and in turn, better knowledge and/or skills that bring them closer to the Mediterranean cultural heritage [77].

The extrapolation of “what” and “how” the families ate to what the adolescents ate individually is another limitation. This is less likely to be the case for younger adolescents than it is for older adolescents, since the latter may have more freedom to choose what they eat and less parental control [78]. Additionally, since this study assessed dietary habits in the family unit, gender and age difference were not studied. Furthermore, possible biases in self-reported food may have occurred.

Another limitation is that most of the interviewed participants were women, who may have a different view from the other parent. Even today, women play a greater role in taking care of meal planning [79], and they seem to be in charge of creating conviviality [8]. Gender differences in perceptions and habits have been observed [78].

One more limitation is that the Mediterranean diet pyramid score has not yet been validated. Nevertheless, the MEDAS is a validated measure, and there was a significant correlation between the Mediterranean diet pyramid score and MEDAS, suggesting that both could be good “a priori” methods for assessing the families’ dietary patterns.

A strength of this qualitative study is that it enabled families to voice their views and experiences of conviviality. The interviews allowed the parents to speak openly and freely, using their own words and with no restrictions imposed on their responses. Furthermore, the use of qualitative methods allowed for gaining an in-depth and comprehensive understanding of the meanings given by Mediterranean families to conviviality, which would not have been possible using only a quantitative methodology [80].

**Figure 1:**
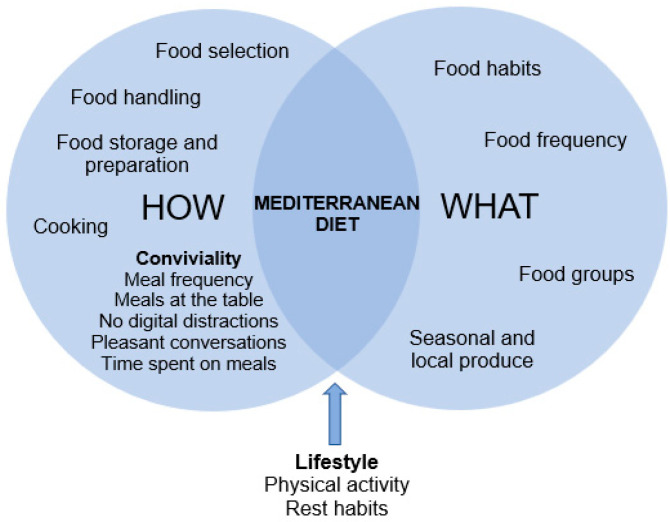
The “what” and “how” of the Mediterranean diet.

Future longitudinal studies are needed to examine the phenomenon of family conviviality within Mediterranean contexts in larger samples of families with different socioeconomic levels and weight statuses and its effect on the dietary patterns and health of families. It is not only the nutritional quality of the meal that must be considered to have healthy family meals. Interventions to exclusively promote family meals are needed to further explore causality between conviviality and health and wellbeing outcomes. Figure 1 shows the “what” and “how” of the MD, which includes conviviality as a component [4].

## 5. Conclusions

In conclusion, since there have been few studies on conviviality in the Mediterranean area and they have mainly been theoretical in nature, this study moves forward our understanding of the “how” of the MD. The study analyzed the practices and representations of family meals that are associated with a conviviality pattern and their relationship with eating habits. Items relating to the conviviality of family meals identified in the study were meal frequency, meals at the table, lack of digital distractions, pleasant conversations, and time spent on family meals. The results do seem to support that families with a less clear pattern of conviviality (i.e., spent less time on family meals, meals were not at the table, had digital distractions, did not enjoy meals through pleasant conversations) have a lower MD adherence, whereas six of the nine families with high adherence fulfilled all the conviviality items. The findings showed that parents believed family meals to be a space for socialization and communication and that parents act as role models for adolescents’ food preferences. Dinner was considered the family meal par excellence among the interviewed families with optimal family meal frequency (≥ 5/week). Family and non-family meals often differed in their food composition, location, use of technology, and duration.

Furthermore, conviviality is an element of the MD as an intangible cultural heritage, which relates to the pleasure associated with eating together, or a particular attitude towards shared meals. There is considerable scientific evidence of the health benefits of the MD in general in terms of its food components [1], but there has been little exploration of how each of its components, including the cultural aspect inherent to the MD pattern, are important in terms of health promotion for families with adolescents. Indeed, the MD, with its conviviality elements, could be a way to better trigger the satiety response and indirectly prevent childhood obesity [68]. More attention should be paid to conviviality in families in Mediterranean areas when designing multi-approach strategies to promote healthy eating among adolescents, with families being the target for the preservation and transmission of the MD to the younger generations.

## Figures and Tables

**Table 1:** Characteristics of the participants.

Family	Socioeconomic Status ^1^	Parent Interviewed	Mother’s Education	Father’s Education	Mother’s Employment	Father’s Employment	Number of Adolescents in Household	Adolescents’ Gender ^2^	Adolescents’ Age ^2^	Adolescents’ Weight Status ^3^
1	High	Mother	University	University	Part-time	Full-time	1	M	14	Underweight
2	Medium	Mother	Secondary	Secondary	Part-time	Full-time	2	F/F	14/14	Overweight/ Normal
3	High	Mother	University	Secondary	Full-time	Full-time	1	M	14	Normal
4	High	Mother	University	University	Full-time	Full-time	2	F/M	14/16	Normal/Normal
5	High	Mother	University	University	Full-time	Full-time	1	F	14	Normal
6	High	Mother and Father	University	Secondary	Full-time	Full-time	1	M	16	Normal
7	High	Mother	University	University	Full-time	Full-time	1	M	16	Normal
8	High	Mother	University	University	Full-time	Full-time	1	M	16	Normal
9	High	Mother	University	University	Full-time	Full-time	1	F	16	Normal
10	High	Mother	University	University	Full-time	Full-time	1	F	15	Normal
11	Medium	Mother	Secondary	Secondary	Full-time	Leave of absence	3	F/M/M	14/14/14	Normal/Normal/Normal
12	High	Mother and Father	Secondary	Primary	Full-time	Full-time	1	F	14	Normal

^1^ A weighted average of each parent’s education and employment level based on Hollingshead’s (1975) “Four-Factor Index of Social Status” was calculated to determine the socioeconomic status. ^2^ Information about the participants’ gender and age is presented from the youngest to the eldest child. ^3^ We used the World Health Organization (WHO) cut-off values of the Body Mass Index (BMI) for ages 5–19 years to determine body mass index (BMI). Interpretation of the cut-offs: overweight: ≥1SD (equivalent to BMI ≥ 25 kg/m^2^ at 19 years), obesity: ≥2SD (equivalent to BMI ≥ 30 kg/m^2^ at 19 years), underweight: ≤2SD.

**Table 2:** Themes from the thematic analysis of the interviews.

Topic	Themes	Definition of the Theme
Parents’ representations of family meals	Theme 1: Importance of family meals and the interactions they produce	Explanations of what family meals and the interactions they produce (including conversations) mean to them
	Theme 2: Parents’ influence on adolescents’ food preferences	Explanations of how family meals can influence children’s and adolescents’ food preferences
Practices in family meals	Theme 3: Daily meal routines	Explanations of the families’ mealtime routines including meal frequency
	Theme 4: Location of meals	Explanations of the spaces where meals are eaten
	Theme 5: Use of technologies	Explanations of the use of screens (TV, tablets, mobiles) during mealtimes
	Theme 6: Communication	Explanations of the themes and dynamics of mealtime conversations
	Theme 7: Organization	Explanations of the family’s aspects related to the organization of meals
	Theme 8: Meal duration	Explanations of meal duration and the difference between the duration of family and non-family meals
	Theme 9: Rules	Explanations of the rules during family meals
	Theme 10: Dietary habits	Parents’ explanations of aspects related to their dietary habits in family and non-family meals

**Table 3:** Results of Mediterranean diet adherence, food habits, and conviviality practices.

Groups According to Adherence	Very Optimal Adherence				Optimal Adherence					Low Adherence		
Family identification number	01	04	07	11	05	06	09	10	12	02	03	08
Mediterranean diet pyramid score	32	34	31	32	28	30	29	30	29	22	26	26
Ultra-processed food consumption (1)	1	0	1	0	2	0	2	0	0	2	1	1
Family meal frequency (2)	X	X	X	X	X	X	X	X	X	X	X	X
Meals at the table (3)	X	X	X	-	X	-	X	X	X	X	X	-
No digital distractions (4)	X	X	X	-	X	-	X	X	X	-	X	-
Pleasant conversations (5)	X	-	X	X	X	X	X	X	X	-	X	X
Meal duration (6)	X	X	X	X	X	X	X	X	X	X	-	-

X means that the item was accomplished, and the hyphen means that it was not. Supplementary Table S2 describes how the Mediterranean diet pyramid score was built and its categories. Source: FFQ. (1) Weekly portions of ultra-processed food. “0” means no consumption; “1”, medium consumption; “2”, high consumption. Source: FFQ, interview, and photos. (2) More than 5 family meals per week. Source: Family meal questionnaire. (3) Families had their meals at the kitchen or dining-room table. Not on the sofa. Source: Interview. (4) No screens (TV or mobile phone) are actively used or are often used during meals. Source: FFQ and interview. (5) Parents reported that mealtime conversations were pleasant. Source: interview. (6) Families spent more than 30 min on family meals. Source: FFQ.

## Data Availability

The data presented in this study are available on request from the corresponding author. The data are not publicly available due to privacy or ethical reason.

## Supplementary Materials

The following are available online at https://www.mdpi.com/1660-4601/18/5/2499/s1, Table S1: Topics and questions of the parents’ interview, Table S2: Mediterranean diet score, Table S3: Results of Mediterranean diet adherence scores, Figure S1: Composition of the interviewed families’ dinners in comparison to the Harvard Healthy Eating Plate.
