# Supplementary material for: Family Meals, Conviviality, and the Mediterranean Diet among Families with Adolescents
Source: Int J Environ Res Public Health. 2021 Mar 3;18(5):2499. doi: 10.3390/ijerph18052499 (PMC7967627; doi:10.3390/ijerph18052499)
Supplement: Supplementary file 1 [file ijerph-18-02499-s001.pdf]

Supplementary Material

Table S1. Parents' interview topics and questions.

Topics	Questions
Family activities	What is the family's daily routine?<br>What meals do you usually have as a family?
Location of family meals	Where are family meals usually eaten?<br>Is the location the same when it is a non-family meal?
Importance of family meals	Do you think family meals are important? Why?
Use of technology	What do you think about the use of screens (TV, tablets, mobile phones) at family mealtimes?<br>Is the use of screens allowed during family meals? Why/Why not?<br>Is the use of screens allowed when it is a non-family meal? Why/Why not?
Conversations	What do you usually talk about at family meals?<br>How or who usually starts these conversations?<br>How important are these conversations to you?
Meal organization	Who cooks the family meals? Why/Why not?<br>How do you decide what to eat?<br>How do you ensure the food is ready at mealtimes?<br>Do you have an assigned place at the table for meals? Why/Why not?<br>How and who serves up the food?<br>Are there any rules for mealtimes? For example, not getting up until everyone has finished.<br>Is there a difference between the time spent on having family and non-family meals? Why?
Dietary habits	Decribe a typical family meal menu.<br>How does the menu change if the meal is a non-family meal? Do the amounts of food change as well? Why?<br>How do you think family meals affect how and what you eat?<br>How do you think family meals affect your children's food preferences?<br>Are there different food preferences? Does everyone eat the same thing? Why?<br>How do you think different food preferences affect the preparation and organization of family meals?

Table S2. Mediterranean diet pyramid score.

Items	Sub-items	Score
Priority water consumption	Priority water consumption	1
	No priority water consumption	0
Fruit at each main meal	Fruit at each main meal	2
	Fruit at one meal	1
	Fruit at no meal	0
Vegetables at each main meal	Vegetables at each main meal	2
	Vegetables at one meal	1
	Vegetables at no meal	0
Cereals at each main meal	Cereals at each main meal	2
	Cereals at one meal	1
	Cereals at no meal	0
Priority whole grain consumption	Priority whole grain consumption	1
	No priority whole grain consumption	0
Priority olive oil consumption	Priority olive oil consumption	1
	No priority olive oil consumption	0
Seasoning with spices and garlic	Seasoning with spices and garlic	1
	No seasoning with spices and garlic	0
Nut consumption	1 handful of nuts daily	3
	4–6 handfuls of nuts weekly	2
	1–3 handfuls of nuts weekly	1
	No consumption of nuts	0
Dairy consumption	2 portions of dairy daily	2
	1 portion of dairy daily	1
	No consumption of dairy	0
Fish and seafood consumption	> 2 portions of fish and seafood weekly	2
	1 portion of fish and seafood weekly	1
	No consumption of fish and seafood	0
White meat consumption	2 portions of white meat weekly	2

	1 portion of white meat weekly	1
	No consumption of white meat	0
Legume consumption	> 2 portions of legumes weekly	2
	1 portion of legumes weekly	1
	No consumption of legumes	0
Egg consumption	3 portions of eggs weekly	3
	2 portions of eggs weekly	2
	1 portion of eggs weekly	1
	No consumption of eggs	0
Processed meat consumption	No consumption of processed meat	3
	1 portion of processed meat weekly	2
	2 portions of processed meat weekly	1
	> 3 portions of processed meat weekly	0
Red meat consumption	< 2 portions of red meat weekly	2
	3 portions of red meat weekly	1
	> 4 portions of red meat weekly	0
Potato consumption	≤ 2 portions of potatoes weekly	2
	3 portions of potatoes weekly	1
	> 4 portions of potatoes weekly	0
Pastry and sweet consumption	No consumption of pastries and sweets	3
	1 portion of pastries and sweets weekly	2
	2 portions of pastries and sweets weekly	1
	> 3 portions of pastries and sweets weekly	0
Home-cooked food	High consumption of home-cooked food	3
	Medium consumption of home-cooked food	2
	Low consumption of home-cooked food	1
	No consumption of home-cooked food	0
Prioritization of family meals	Prioritization of family meals	2
	Some prioritization of family meals	1
	No prioritization of family meals	0
**Classification according score**		**Points**
Very low Mediterranean diet adherence		0–12
Low Mediterranean diet adherence		13–26
High Mediterranean diet adherence*		27–39

*Two subcategories were developed to further stratify high adherence families: "very optimal adherence" (39–31) and "optimal adherence" (30–27).

Table S3. Results of Mediterranean diet adherence scores.

MD adherence scores	***Family identification number***											
	01	*02*	*03*	*04*	*05*	*06*	*07*	*08*	*09*	*10*	*11*	*12*
Mediterranean diet pyramid score (1)	*32*	*22*	*26*	*34*	*28*	*30*	*31*	*26*	*29*	*30*	*32*	*29*
MEDAS* (2)	*11*	*7*	*9*	*13*	*10*	*10*	*10*	*9*	*10*	*11*	*11*	*11*

(1). Source: FFQ. Supplementary Table 2 describes how the Mediterranean diet pyramid score was built and its categories. (2). Source: FFQ * Schröder, H.; Fitó, M.; Estruch, R. A Short Screener Is Valid for Assessing Mediterranean Diet Adherence among Older Spanish Men and Women. *J. Nutr.* **2011**, *141* (6), 1140–1145. MEDAS: Mediterranean diet adherence screener.

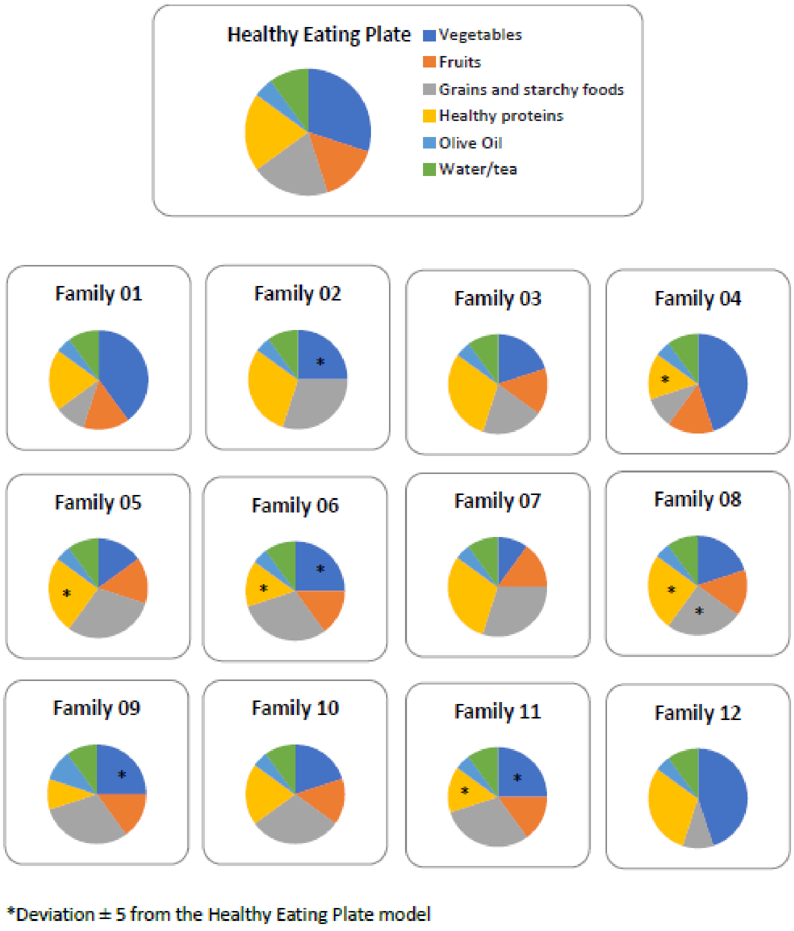

Figure S1. Composition of the interviewed families' dinners in comparison to the Harvard Healthy Eating Plate (*The Healthy Eating Plate. Harvard University, 2011).*
